# A Girl with Cutaneous Lesions, Polyarthritis, and Antinuclear Antibodies Positivity

**DOI:** 10.5402/2011/657673

**Published:** 2011-04-04

**Authors:** Jorge L. Musuruana, Javier A. Cavallasca

**Affiliations:** Section of Rheumatology and Autoimmune Diseases, Hospital JB Iturraspe, Bv. Pellegrini 3551, 3000 Santa Fe, Argentina

## Abstract

On October 1996, a 14-year-old girl was admitted to the hospital because cutaneous lesions, asthenia, and arthralgias. On examination, there was nonscarring hair thinning with a widened part over the frontal hairline, polymorphic papulosquamous rash on her face, neck, arms, and trunk, and livedo reticularis in her legs. Multiple aphtous ulcers were present on the buccal and nasal mucosa. There was polyarthritis involving the wrist, metacarpophalangeal joints, proximal interphalangeal joints, and metatarsophalangeal joints of both hands and feet. Skin biopsy of the face was compatible with subacute cutaneous lupus erythematosus. She started on prednisone 60 mg/d without improvement, and later hdroxhchloroquine (HCQ) 6 mg/kg/d was added for one year. Cutaneous lesions were almost healed, with just a hypopigmented macules left. Over the last 14 years, she has not shown any cutaneous or systemic manifestations.

## 1. Case Report

On October 1996, a 14-year-old girl was admitted to the hospital because of cutaneous lesions, asthenia, and arthralgias. She had been in good health until four months earlier, when she began to experience asthenia, decreased appetite, photosensitivity, hair loss, erythematous papules on her face, and diffuse arthralgias. On examination, the patient's blood pressure was 110/60 mm Hg, her temperature was 36,8°C, her pulse rate was 68 beats/min, and her respiration rate was 16 breaths/min. 

**Figure 1:**
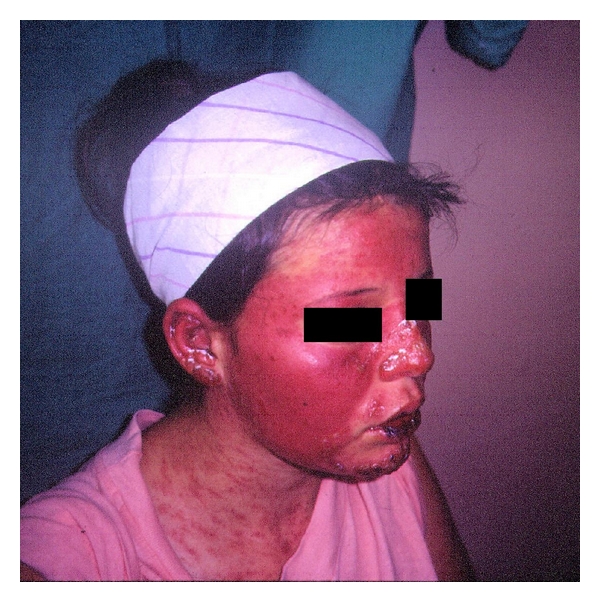
Photosensible malar rash with multiple, symmetric, red to violaceous slightly scaly confluent papules and plaques. Some of the small papules became confluent to form large plaques.

**Figure 2:**
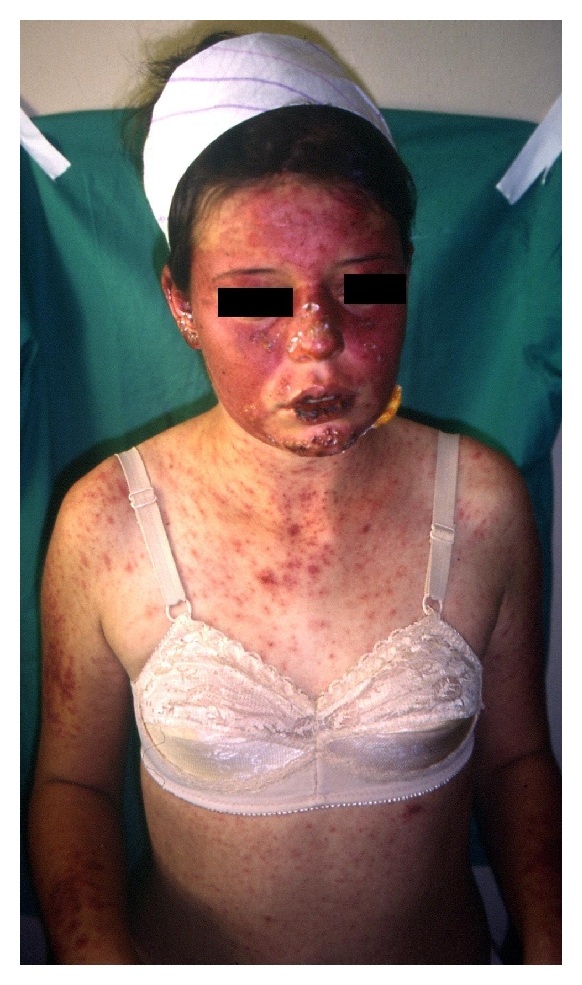
Multiple red to violaceous confluent papules and plaques on the face, trunk, arms, and abdomen.

There was nonscarring hair thinning with a widened part over the frontal hairline, polymorphic papulosquamous rash on her face, neck, arms, and trunk, (Figures 1 and 2), and livedo reticularis in her legs. Multiple aphtous ulcers were present on the buccal and nasal mucosa. There was polyarthritis involving the wrist, metacarpophalangeal joints, proximal interphalangeal joints, and metatarsophalangeal joints of both hands and feet. The rest of the physical examination findings were normal.

A complete blood count with differential analysis, liver function tests, chemistry panel, muscle enzymes, and urinalysis was normal. A test for antinuclear antibodies (ANA) was positive at a titer of 1 : 160 with a homogeneous-speckled pattern, but the remainder of the antinuclear-antibody screening (anti-double-stranded DNA, anti-Ro/SSA, anti-La/SSB, and anti ENA) was negative. An antineutrophil cytoplasmic antibody (ANCA) test, rheumatoid factor, anticardiolipin antibodies Ig G and Ig M, VDRL, and HIV were all negative. Complement levels (C3, C4, and CH50) were within normal limits. 

A chest X-ray, an electrocardiogram, an transthoracic echocardiography, and an abdominal US were normal. 

**Figure 3:**
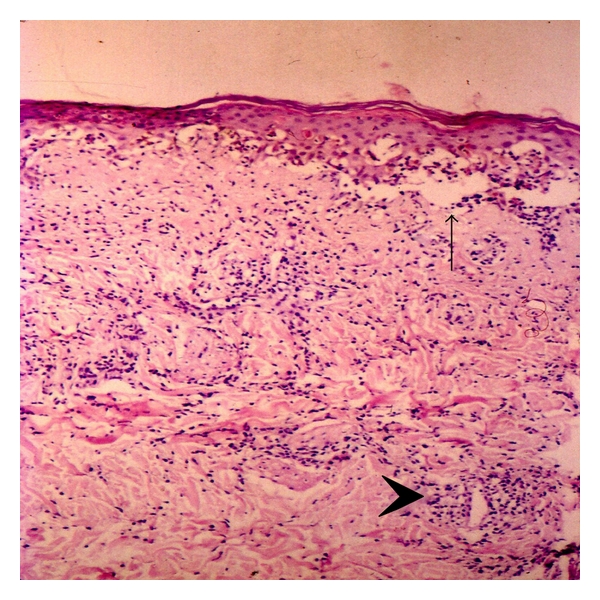
Cheek biopsy showing epidermal atrophy, vacuolar interface dermatitis (arrow) with necrotic keratinocytes in the epidermis, and a perivascular infiltrate with lymphocytes and macrophages (arrowhead).

Skin biopsy of the face was compatible with subacute cutaneous lupus erythematosus (Figure 3).

She started with prednisone 60 mg/d without improvement; after two months this medication was discontinued. Later hydroxychloroquine (HCQ) 6 mg/kg/d was added for one year, then, the dose was tapered to 3 mg/kg/d. The patient had an extraordinary response to HCQ. Cutaneous lesions were almost healed, with just a hypopigmented macules left. Over the last 14 years, she has not shown any cutaneous or systemic manifestations.

## 2. Discussion

The skin lesions seen in patients with lupus can be classified into those that are lupus-specific histologically and those that are lupus-nonspecific. There are three broad categories of LE-specific skin disease: acute cutaneous LE (ACLE), subacute cutaneous LE (SCLE), and chronic cutaneous LE (CCLE) [1]. 

Subacute cutaneous lupus erythematosus is a photosensitive, superficial nonscarring type of cutaneous LE, with lesions being seen most commonly on the V area, upper back, shoulders, extensor surfaces of the arms and forearms, and dorsum of the hands [2]. SCLE has two clinical forms: annular SCLE and papulosquamous SCLE [1, 2]. Our patient presented papulosquamous lesions. 

During the cutaneous outbreak, patients often have systemic manifestations, particularly at the articular level, as it happened with our patient. 

Approximately 70% of all SCLE patients have Ro/SSA (+) [1]. Nonspecific LE skin lesions such as vasculitis, livedo reticularis, and alopecia are frequently seen in patients with cutaneous LE [3]. 

About half of all patients with SCLE fulfil four or more of the American College of Rheumatology (ACR) criteria for systemic lupus erythematosus (SLE) but have been noted to have a mild subset of systemic disease[2]. Our patient has five ACR criteria for SLE classification (malar rash, photosensitivity, oral ulcers, arthritis, and ANA positivity).

The histologic hallmark of LE-specific skin lesions is the vacuolar degeneration in the basal layer of the epidermis (hydropic or liquefactive degeneration) [1].

Sunscreens are a cornerstone of therapy and they should be applied daily to all exposed surfaces. Low-to-medium potency topical steroids should be used to treat facial lesions and trunk and arm lesions with medium-potency agents, and lesions on the palms and soles should be treated with high-potency topical glucocorticoids. Systemic corticosteroids are partially effective for SCLE lesions; however, because of the delayed onset of action of antimalarials, there may be indicated despite their potential severe side effects, such as avascular necrosis, hypeglycemia, or osteoporosis [4, 5] .

The most useful systemic agents for treating LE-specific skin lesions are the aminoquinoline antimalarial agents. They have multiple sun blocking, anti-inflammatory and immunosuppressive effects. Antimalarials occrue in different concentrations among the various body tissues and organs. The highest of all concentrations occurs in melanin-containing cells such as found in the retina and skin. Hydroxychloroquine is the initial treatment of choice. Its onset of action is approximately 1 month, even though its full benefit might not be seen for several months. It is typically dosed at 200 or 400 mg per day and should not exceed 6.5 mg/kg/d. At these doses, it is usually well tolerated. Chloroquine is more toxic and it is used in a 4 mg/kg/d dose. Quinacrine does not cause retinal toxicity, and thus can be used in combination with either hydroxychloroquine or chloroquine [6]. Approximately 75% of SCLE patients improved with antimalarials agents, smokers are less likely to do so [5]. 

Other treatments include thalidomide, topical tacrolimus, dapsone, gold salts, oral retinoids, and oral fenitoin. Cytotoxic immunosuppressive agents, such as azathioprine, mycophenolate mofetil, and methotrexate are reserved for patients with more severe disease who have failed the less toxic forms of therapy [4].
